# GAOC: A Gaussian Adaptive Ochiai Loss for Bounding Box Regression

**DOI:** 10.3390/s26020368

**Published:** 2026-01-06

**Authors:** Binbin Han, Qiang Tang, Jiuxu Song, Zheng Wang, Yi Yang

**Affiliations:** 1 School of Electronic Engineering, Xi’an Shiyou University, Xi’an 710312, China; hanbinbin@xsyu.edu.cn (B.H.); jxsong@xsyu.edu.cn (J.S.); zhwang@xsyu.edu.cn (Z.W.); 2 University of Chinese Academy of Sciences, Beijing 100049, China; tangqiang22@mails.ucas.ac.cn; 3 Xi’an Institute of Optics and Precision Mechanics of CAS, Xi’an 710119, China

**Keywords:** bounding box regression, ochiai coefficient, Gaussian adaptive distribution, object detection

## Abstract

Bounding box regression (BBR) loss plays a critical role in object detection within computer vision. Existing BBR loss functions are typically based on the Intersection over Union (IoU) between predicted and ground truth boxes. However, these methods neither account for the effect of predicted box scale on regression nor effectively address the drift problem inherent in BBR. To overcome these limitations, this paper introduces a novel BBR loss function, termed Gaussian Adaptive Ochiai BBR loss (GAOC), which combines the Ochiai Coefficient (OC) with a Gaussian Adaptive (GA) distribution. The OC component normalizes by the square root of the product of bounding box dimensions, ensuring scale invariance. Meanwhile, the GA distribution models the distance between the top-left and bottom-right corners (TL/BR) coordinates of predicted and ground truth boxes, enabling a similarity measure that reduces sensitivity to positional deviations. This design enhances detection robustness and accuracy. GAOC was integrated into YOLOv5 and RT-DETR and evaluated on the PASCAL VOC and MS COCO 2017 benchmarks. Experimental results demonstrate that GAOC consistently outperforms existing BBR loss functions, offering a more effective solution.

## 1. Introduction

Object detection, a core computer vision task, is widely applied in autonomous driving, remote sensing, medical image analysis, and security surveillance [1,2]. Its objective is the simultaneous execution of object classification and localization. In this context, the bounding box regression (BBR) loss function is critical. It directly determines localization accuracy by measuring the alignment between predicted and ground truth boxes, thereby influences overall detection performance. Recent advances in deep learning have prompted the development of numerous Intersection over Union (IoU)-based loss functions [3], including GIoU, DIoU, CIoU, and EIoU. These functions mitigate issues such as gradient vanishing for non-overlapping boxes, center-point distance, and aspect ratio mismatch, thereby driving continual improvements in detection accuracy.

However, prevailing IoU-based loss functions suffer from two principal limitations. First, they frequently ignore the influence of bounding box scale on the regression process, resulting in suboptimal performance for small objects and in long-tail distributions. Second, these methods are often ineffective at mitigating the BBR drift problem. This problem arises when a significant positional deviation between the predicted and ground truth boxes prevents the loss function from providing sufficient gradient constraints. This inadequacy can cause slow convergence during initial training or unstable localization in later phases. Consequently, the development of a BBR loss function that ensures both scale invariance and robustness remains a pressing challenge in object detection research.

To overcome these limitations, this paper proposes a novel BBR loss function named Gaussian Adaptive Ochiai Loss (GAOC). GAOC integrates the properties of the Ochiai Coefficient (OC) and a Gaussian Adaptive (GA) distribution. The OC ensures scale invariance through normalization by the square root of the bounding box areas. Concurrently, the GA component models the top-left and bottom-right corners (TL/BR) coordinates with a Gaussian distribution, thereby reducing sensitivity to significant positional deviations. This combined approach enhances the loss function’s adaptability across object scales and improves its robustness and accuracy in complex detection scenarios. We integrated GAOC into the YOLOv5 [4] and RT-DETR [5], evaluate on PASCAL VOC [6,7] and MS COCO 2017 [8]. The results demonstrate that GAOC consistently surpasses existing IoU-based loss functions across multiple evaluation metrics, confirming its strong generality and effectiveness.

The primary contributions of this study are as follows:

- We propose a novel BBR loss based on the Ochiai Coefficient (OC). Compared to traditional IoU loss, OC emphasizes the balance of bounding box dimensions while assigning greater weight to the intersection of the two boxes.
- We introduce a Gaussian adaptive (GA) distribution for BBR loss, which improves robustness to positional variations by modeling TL/BR distances as a two-dimensional Gaussian distribution and computing similarity through GA.
- We validate the effectiveness of GAOC on public datasets, where it outperforms other BBR losses across multiple benchmarks.

The remainder of this paper is organized as follows. Section 2 reviews representative IoU-family bounding box regression losses and discusses their limitations. Section 3 presents the proposed GAOC loss, including its formulation, optimization properties, and implementation details. Section 4 describes the experimental settings and reports comprehensive comparisons and ablation studies on benchmark datasets. Finally, Section 5 concludes this work and outlines future directions.

## 2. Related Work

### 2.1. Object Detection

Object detection is a fundamental task in computer vision, which involves identifying objects in images and accurately determining their locations and categories. R-CNN [9] was a pioneering approach in this field, introducing selective search to generate candidate object regions. These regions were then processed by Convolutional Neural Networks (CNNs) for feature extraction, followed by object classification using Support Vector Machines (SVMs).

Building on R-CNN, a series of improved variants have been developed, including Fast R-CNN [10], Faster R-CNN [11], and Mask R-CNN [12], each achieving significant gains in both efficiency and accuracy. In parallel, the SSD [13] and YOLO [4,14,15,16] families have become widely adopted due to their balance of speed and accuracy, enabling real-time object detection within an end-to-end framework. More recently, algorithms such as CornerNet [17], CenterNet [18], and FCOS [19] have emerged, advancing object detection through novel architectural designs and innovative methodologies.

The DETR [5] family of algorithms, distinguished by its attention mechanism and Transformer-based architecture, introduces a novel paradigm for object detection. Despite their diverse design principles and implementations, object detection algorithms share a common challenge in BBR, a critical step in accurate object localization. Precise bounding box prediction enables these models to localize objects reliably within images, thereby providing a robust foundation for subsequent analysis and processing.

### 2.2. Bounding Box Regression Losses

Existing mainstream BBR losses are primarily built upon the IoU [3], defined as:

(1) $$IoU=\frac{|B\cap B^{gt}|}{|B\cup B^{gt}|},$$

where *B* and $B^{gt}$ denote the predicted and ground truth boxes, respectively. The corresponding loss $L_{IoU}$ is defined as:

(2) $$L_{IoU}=1-IoU.$$

IoU-based losses have become the dominant methodology for BBR.

The GIoU loss [20] mitigates the problem of gradient vanishing during bounding box updates when the predicted boxes and the ground truth are non-overlapping. The GIoU loss is defined as:

(3) $$GIoU=IoU-\frac{\left|C\setminus (B\cap B^{gt})\right|}{\left|C\right|};$$

Here *C* represents the smallest convex hull that encloses both *B* and $B^{gt}$.

In contrast to GIoU, the DIoU [21] loss introduces an additional distance term into the IoU formulation, which minimizes the normalized distance between the centroids of the two bounding boxes, thereby enabling the algorithm to achieve faster convergence and improved performance. The DIoU is defined as:

(4) $$DIoU=IoU-\frac{\rho^{2}\left(b,b^{gt}\right)}{c^{2}},$$

where *b* and $b^{gt}$ represent the centroids of *B* and $B^{gt}$, respectively. The term ρ(·) represents the Euclidean distance, and *c* denotes the diagonal of the smallest enclosing bounding box.

The CIoU [21] loss extends DIoU by incorporating an additional shape loss term that accounts for aspect ratio consistency. The CIoU is formally defined as follows:

(5) $$CIoU=IoU-\frac{\rho^{2}\left(b,b^{gt}\right)}{c^{2}}+\alpha v,$$

where α is the trade-off parameter given by:

(6) $$\alpha =\frac{v}{(1-\mathrm{IoU})+v},$$

and *v* quantifies aspect ratio consistency:

(7) $$v=\frac{4}{\pi^{2}}{\left(\arctan\frac{w^{gt}}{h^{gt}}-\arctan\frac{w}{h}\right)}^{2}.$$

**Figure 1:**
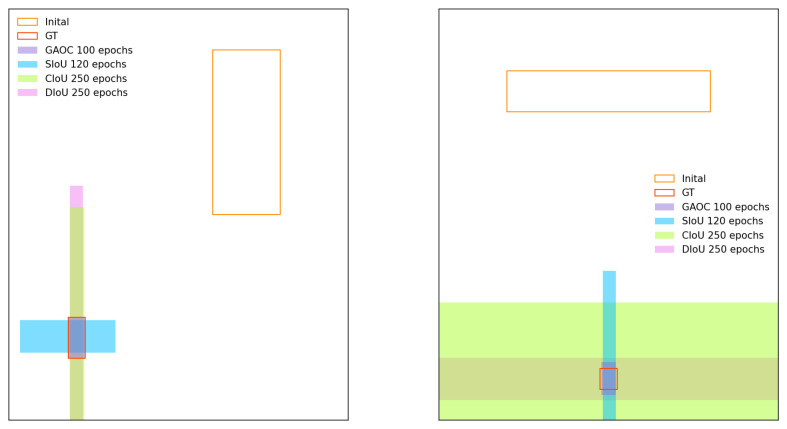
Regression results under various BBR loss functions indicate that GAOC achieves the best overall performance.

Here, $w^{gt}$ and $h^{gt}$ represent the width and height of the ground truth box, while *w* and *h* represent those of the predicted box. When the ground truth and predicted boxes share the same aspect ratio, CIoU simplifies to DIoU. The results of various losses are presented in Figure 1.

The EIoU [22] loss directly minimizes the normalized differences in width $\left(w,w^{gt}\right)$, height $\left(h,h^{gt}\right)$, and centroid position $\left(b,b^{gt}\right)$ between the predicted and ground truth boxes. The EIoU loss is defined as:

(8) $$EIoU=IoU-\frac{\rho^{2}\left(b,b^{gt}\right)}{c^{2}}+\frac{\rho^{2}\left(w,w^{gt}\right)}{{\left(w^{c}\right)}^{2}}+\frac{\rho^{2}\left(h,h^{gt}\right)}{{\left(h^{c}\right)}^{2}},$$

where $w^{c}$ and $h^{c}$ represent the width and height of the smallest enclosing bounding box that contains both the predicted and ground truth boxes.

SIoU [23] extends IoU-based BBR by adding an angle-aware guidance, together with distance and shape penalties, to stabilize optimization and improve localization. It is formulated as

(9) $$SIoU=IoU-\frac{\Delta +\Omega }{2},$$

where Δ and Ω represent the distance and shape costs, respectively, and the distance term is reweighted by an angle-dependent factor.

When the width and height are identical, BBR optimization becomes infeasible. To address this issue and leverage the geometric properties of horizontal rectangles, a bounding box similarity metric, termed minimum-point distance IoU. MPDIoU was proposed [24] and is defined as follows:

(10) $$MPDIoU=\frac{\left|A\cap B\right|}{\left|A\cup B\right|}-\frac{d_{1}^{2}+d_{2}^{2}}{w^{2}+h^{2}},$$

where $d_{1}$ and $d_{2}$ are the distances between the TL and BR corners, respectively, and *w* and *h* denote the width and height of the image.

The WIoU [25] loss is based on a dynamic non-monotonic focusing mechanism. This mechanism utilizes the outlier degree to evaluate the quality of predicted boxes and designs a gradient gain allocation strategy. The strategy reduces the dominance of high-quality predicted boxes while mitigating the adverse gradients from low-quality examples, thereby enabling WIoU to focus on medium-quality predicted boxes and enhance the detector’s overall performance. WIoU is defined as follows:

(11) $$L_{WIoU}=R_{WIoU}\cdot IoU,$$

(12) $$R_{WIoU}=\exp\left(\frac{{\left(x-x_{gt}\right)}^{2}+{\left(y-y_{gt}\right)}^{2}}{{\left(W_{g}^{2}+H_{g}^{2}\right)}^{*}}\right),$$

where $R_{WIoU}\in \left[1,e\right)$ significantly amplifies the $L_{IoU}$ of ordinary-quality predicted boxes ($L_{IoU}\in \left[0,1\right]$). When predicted box and the ground truth overlap well, $L_{IoU}$ significantly reduces $R_{WIoU}$ for high-quality predicted boxes, with a focus on the centroid distance. Here, $W_{g}$ and $H_{g}$ denote the dimensions of the minimum enclosing boxes.

### 2.3. Limitations

Despite substantial progress in IoU-based BBR losses, practical limitations remain. GIoU, DIoU, CIoU primarily improve overlap and center and aspect constraints, but can be less effective under large displacement, extreme aspect ratios, or long-tail scales. EIoU and SIoU further refine geometric penalties, yet introduce additional design complexity and potential sensitivity to hyperparameters. MPDIoU emphasizes point-wise geometry but does not explicitly improve scale robustness, while WIoU mainly reweights samples without changing the underlying overlap similarity, leaving limited guidance when overlap is low. Motivated by these gaps, we propose the GAOC, which enhances scale robustness via Ochiai-style normalization and strengthens geometric guidance through Gaussian-adaptive corner modeling for more stable regression under scale variation and large displacement.

## 3. Method

### 3.1. Simulation Experiment

This study employs a simulation experiment proposed in the CIoU [21] study to evaluate BBR performance under different loss conditions. The simulation generates seven target boxes (aspect ratios 1:4, 1:3, 1:2, 1:1, 2:1, 3:1, and 4:1; all with area 1/32) centered at the coordinate (0.5, 0.5). Twenty thousand anchor points are generated in a circular area of radius *r*, centered at the same location, and for each anchor point, 49 anchor boxes (aspect ratios 1/32, 1/24, 3/64, 1/16, 1/12, 3/32, 1/8) are defined. Each anchor box is regressed toward the target box.

**Figure 2:**
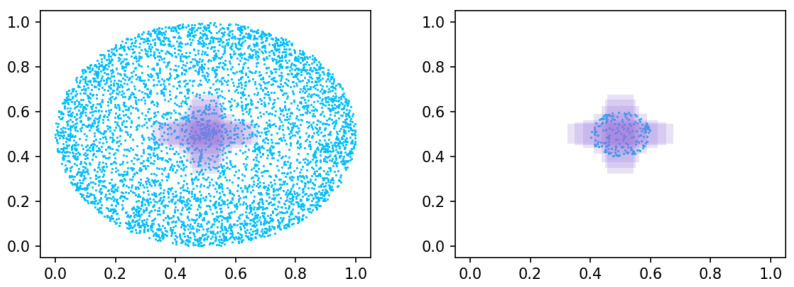
Simulation experiments with anchor points (blue) and object boxes (purple). (**left**) All cases. (**right**) Major cases.

To compare convergence speeds at different stages, this study adopts the following experimental settings: for r=0.5, anchor points are distributed both inside and outside the target box (Figure 2, left), representing the full range of BBR scenarios; for r=0.1, anchor points lie within the target box (Figure 2, right), representing the primary BBR scenarios.

### 3.2. Ochiai Coefficient Loss

#### 3.2.1. Loss Forward

**Figure 3:**
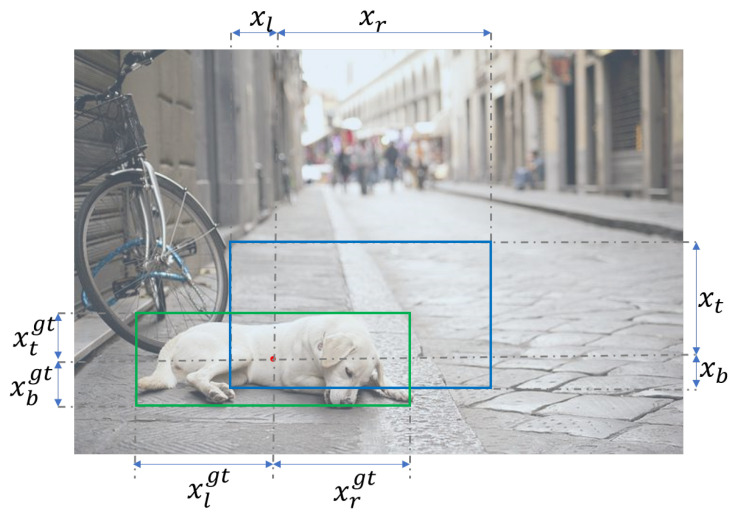
Illustration of OC loss for pixel-wise predicted box.

To overcome the scale sensitivity of IoU-style normalization, this study proposes a novel Ochiai Coefficient (OC) loss, defined as follows. For each pixel p(i,j) in the image, the ground truth is represented as a four-dimensional vector: $x_{i,j}^{gt}=\left(x_{ij}^{t},x_{ij}^{b},x_{ij}^{l},x_{ij}^{r}\right),$ where $x_{t}$, $x_{b}$, $x_{l}$, and $x_{r}$ represent the distances from pixel (i,j) to the top, bottom, left, and right boundaries of the image, respectively. The predicted box is similarly defined as $x=(x_{t},x_{b},x_{l},x_{r})$, as illustrated in Figure 3.

For OC loss, given a predicted box $B=(x_{t},x_{b},x_{l},x_{r})$ and a ground truth $B^{gt}$, the loss is defined as:

(13) $$OC=\frac{|B^{gt}\cap B|}{\sqrt{|B^{gt}\left|\times |B\right|}}.$$

In Algorithm 1, p(i,j) indicates whether pixel (i,j) lies within a valid target box. $x^{p}$ and $x^{gt}$ represent the areas of the predicted and ground truth boxes, respectively. $I_{h}$ and $I_{w}$ represent the height and width of the intersection region *I*. Since 0≤OC≤1, we adopt the negative log-likelihood L=−ln(OC). This formulation yields a scale invariant similarity that emphasizes the intersection while balancing box sizes, thereby facilitating more accurate bounding box prediction.

Moreover, this definition naturally normalizes OC to [0,1] independently of the bounding box scale. OC loss emphasizes the weight of shared elements between boxes by considering the balance between predicted box and ground truth sizes. Finally, OC accounts for set size by normalizing with the square root of the product, which renders it invariant to set size.
**Algorithm 1** OC loss Forward  1:**Input:** $x^{gt}$ as ground truth, $x^{p}$ as predicted  2:**Output:** *L* as localization error each pixel (i,j) $x^{gt}\neq 0$  3:$X=\left(x_{t}+x_{b}\right)\cdot \left(x_{l}+x_{r}\right)$  4:$\tilde{X}=\left({\tilde{x}}_{t}+{\tilde{x}}_{b}\right)\cdot \left({\tilde{x}}_{l}+{\tilde{x}}_{r}\right)$  5:$I_{h}=\min\left(x_{t},{\tilde{x}}_{t}\right)+\min\left(x_{b},{\tilde{x}}_{b}\right)$  6:$I_{w}=\min\left(x_{l},{\tilde{x}}_{l}\right)+\min\left(x_{r},{\tilde{x}}_{r}\right)$  7:$I=I_{h}\cdot I_{w}$  8:$U=X\times \tilde{X}$  9:$OC=\frac{I}{\sqrt{U}}$10:L=−ln(OC)11:if not valid then, L=0

#### 3.2.2. Loss Backward

For the OC loss backpropagation, the partial derivative of *X* with respect to *x* (denoted $\nabla_{x}X$, where $x\in x_{t},x_{b},x_{l},x_{r}$) is computed first:

(14) $$\frac{\partial X}{\partial x_{t};\left(\mathrm{or}\;\partial x_{b}\right)}=x_{l}+x_{r},$$

(15) $$\frac{\partial X}{\partial x_{l};\left(\mathrm{or}\;\partial x_{r}\right)}=x_{t}+x_{b}.$$

Next, the partial derivative of *I* with respect to *x* (denoted $\nabla_{x}I$) is derived:

(16) $$\frac{\partial I}{\partial x_{t};\left(\mathrm{or}\;\partial x_{b}\right)}=\left\{\begin{array}{cc} I_{w}, & \mathrm{if}\;x_{t}<x_{t}^{\mathrm{gt}}\;\mathrm{or}\;x_{b}<x_{b}^{\mathrm{gt}},\\ 0, & \mathrm{otherwise}, \end{array}\right.$$

(17) $$\frac{\partial I}{\partial x_{l};\left(\mathrm{or}\;\partial x_{r}\right)}=\left\{\begin{array}{cc} I_{h}, & \mathrm{if}\;x_{l}<x_{l}^{\mathrm{gt}}\;\mathrm{or}\;x_{r}<x_{r}^{\mathrm{gt}},\\ 0, & \mathrm{otherwise}. \end{array}\right.$$

Finally, the gradient of the OC localization loss L with respect to *x* is given by:

(18) $$\frac{\partial L}{\partial x}=\frac{I\nabla_{x}X}{U^{2}\mathrm{OC}}=\frac{\nabla_{x}X}{2U}-\frac{\nabla_{x}I}{I}.$$

The OC loss mechanism is best understood through its mathematical formulation: $\nabla_{x}X$ represents the penalty term associated with the predicted box, which is directly proportional to the loss gradient; $\nabla_{x}I$ represents the penalty term associated with the intersection region, which is inversely proportional to the loss gradient. Consequently, minimizing the OC loss requires maximizing the intersection region while simultaneously minimizing the predicted box volume. The limiting case arises when the intersection region equals the predicted box, yielding perfect bounding box alignment.

### 3.3. Gaussian Adaptive Loss

**Figure 4:**
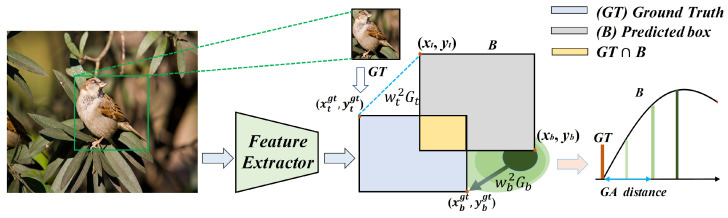
The process of GA. In GA, we directly calculate the GA distance between predicted box and ground truth.

During training, the predicted box B=(x,y,w,h) in Algorithm 2 is optimized to minimise the loss with respect to the ground truth $G=(x_{1},y_{1},w_{1},h_{1})$. Specifically, the TL and BR coordinates of the predicted box *B* are obtained through a transformation $\left(x_{t},y_{t},x_{b},y_{b}\right)$, while the ground truth $G^{gt}$ is defined by the coordinates $\left(x_{t}^{gt},y_{t}^{gt},x_{b}^{gt},y_{b}^{gt}\right)$. Predicted boxes are clustered around the ground truth, with the distance from each predicted box to the ground truth modelled as a Gaussian distribution (GA), as illustrated in Figure 4.
**Algorithm 2** GAOC as Bounding Box Loss  1:**Input** Predicted $B^{p}=\left(x_{t}^{p},y_{t}^{p},x_{b}^{p},y_{b}^{p}\right)$, ground truth $B^{gt}=\left(x_{t}^{gt},y_{t}^{gt},x_{b}^{gt},y_{b}^{gt}\right)$, width and height of input image: w,h.  2:**Output** $L_{GAOC}$  3:For the predicted box $B^{p}$, ensuring $x_{t}^{p}<x_{b}^{p}$ and $y_{t}^{p}<y_{b}^{p}$.  4:$w_{t}^{2}={\left(x_{t}^{p}-x_{t}^{gt}\right)}^{2}+{\left(y_{t}^{p}-y_{t}^{gt}\right)}^{2}$  5:$w_{b}^{2}={\left(x_{b}^{p}-x_{b}^{gt}\right)}^{2}+{\left(y_{b}^{p}-y_{b}^{gt}\right)}^{2}$  6:$G_{t}=\exp\left[-\frac{1}{2}\left(\frac{{\left(x_{t}^{p}-x_{t}^{gt}\right)}^{2}}{2}+\frac{{\left(y_{t}^{p}-y_{t}^{gt}\right)}^{2}}{2}\right)\right]$  7:$G_{b}=\exp\left[-\frac{1}{2}\left(\frac{{\left(x_{b}^{p}-x_{b}^{gt}\right)}^{2}}{2}+\frac{{\left(y_{b}^{p}-y_{b}^{gt}\right)}^{2}}{2}\right)\right]$  8:Calculating area of $B^{gt}:A^{gt}=\left(x_{b}^{gt}-x_{t}^{gt}\right)\cdot \left(y_{b}^{gt}-y_{t}^{gt}\right)$  9:Calculating area of $B^{prd}:A^{p}=\left(x_{b}^{p}-x_{t}^{p}\right)\cdot \left(y_{b}^{p}-y_{t}^{p}\right)$10:Calculating intersection *I* between $B^{prd}$ and $B^{gt}$:11:$x_{1}^{I}=\max\left(x_{t}^{p},x_{t}^{gt}\right),x_{2}^{I}=\min\left(x_{b}^{p},x_{b}^{gt}\right)$12:$y_{1}^{I}=\max\left(y_{t}^{p},y_{t}^{gt}\right),y_{2}^{I}=\min\left(y_{b}^{p},y_{b}^{gt}\right)$13:$I=\left\{\begin{array}{ll} \left(x_{2}^{I}-x_{1}^{I}\right)\cdot \left(y_{2}^{I}-y_{1}^{I}\right), & \mathrm{if}\;x_{2}^{I}>x_{1}^{I},y_{2}^{I}>y_{1}^{I}\\ 0, & \mathrm{otherwise} \end{array}\right.$14:$OC=\frac{I}{\sqrt{B^{gt}\times B^{p}}}$15:$GA=w_{b}^{2}G_{b}+w_{t}^{2}G_{t}$16:GAOC=OC−GA17:$L_{GAOC}=1-GAOC$

Consider a two-dimensional Gaussian distribution for the top-left corner coordinates of the predicted and ground truth boxes, denoted $G_{t}∼N\left(\mu_{1},\mu_{2},\sigma_{1},\sigma_{2},\rho \right)$, where the mean vector is $\mu_{t}=\left[\begin{array}{c} \mu_{1}\\ \mu_{2} \end{array}\right]=\left[\begin{array}{c} x_{t}^{gt}\\ y_{t}^{gt} \end{array}\right]$ The vectors (x,y) are mutually orthogonal, thus the correlation coefficient is ρ=0. Treating the x-axis and y-axis distances as equivalent, the variance matrix $\Sigma_{t}$ is given by:

(19) $$\Sigma_{t}=\left[\begin{array}{cc} \sigma_{1}^{2} & 0\\ 0 & \sigma_{2}^{2} \end{array}\right]=\left[\begin{array}{cc} 2 & 0\\ 0 & 2 \end{array}\right].$$

The two-dimensional Gaussian distribution for distance is:

(20) $$G_{t}={\left(2\pi \sigma_{1}\sigma_{2}\right)}^{-1}\exp\left[-\frac{1}{2}\left(\frac{{\left(x_{t}^{gt}-x_{t}\right)}^{2}}{\sigma_{1}^{2}}+\frac{{\left(y_{t}^{gt}-y_{t}\right)}^{2}}{\sigma_{2}^{2}}\right)\right],$$

(21) $$w_{t}^{2}={\left|x_{t}^{gt}-x_{t}\right|}_{2}^{2}+{\left|y_{t}^{gt}-y_{t}\right|}_{2}^{2},$$

where $w_{t}^{2}$ is the squared Euclidean distance between the TL corners of the predicted and ground truth boxes.

Similarly, the squared Euclidean distance between the BR corners of the predicted and ground truth boxes is:

(22) $$w_{b}^{2}={\left|x_{b}^{gt}-x_{b}\right|}_{2}^{2}+{\left|y_{b}^{gt}-y_{b}\right|}_{2}^{2},$$

with the corresponding Gaussian distribution:

(23) $$G_{b}={\left(2\pi \sigma_{3}\sigma_{4}\right)}^{-1}\exp\left[-\frac{1}{2}\left(\frac{{\left(x_{b}^{gt}-x_{b}\right)}^{2}}{\sigma_{3}^{2}}+\frac{{\left(y_{b}^{gt}-y_{b}\right)}^{2}}{\sigma_{4}^{2}}\right)\right].$$

Here, the values of $\sigma_{3}^{2}$ and $\sigma_{4}^{2}$ are both set to 2, yielding the GA loss:

(24) $$GA=w_{t}^{2}G_{t}+w_{b}^{2}G_{b}.$$

The backward of the GA term can be expressed as:

(25) $$\frac{\partial GA}{\partial (x,y)}=\frac{\partial w_{t}^{2}G_{t}}{\partial (x_{t},y_{t})}+\frac{\partial w_{b}^{2}G_{b}}{\partial (x_{b},y_{b})}=w_{t}^{2}\exp\left(1-\frac{w_{t}^{2}}{2\sigma^{2}}\right)+w_{b}^{2}\exp\left(1-\frac{w_{b}^{2}}{2\sigma^{2}}\right)$$

The GAOC loss is formulated as:

(26) GAOC=OC−GA.

Thus, the $L_{GAOC}$ loss is expressed as:

(27) $$L_{GAOC}=1-GAOC.$$

## 4. Experiments

The objective of this study is to enhance the ability of object detection algorithms to accurately identify objects of diverse sizes and shapes. To this end, we propose the Gaussian Adaptive BBR Loss (GAOC), a novel loss function specifically designed to optimize localisation and classification in object detection. By integrating GAOC into the YOLOv5 and RT-DETR object detection frameworks and conducting extensive experiments on the PASCAL VOC and MS COCO 2017 benchmark datasets, we demonstrate that GAOC achieves superior performance in multi-scale object detection tasks.

### 4.1. MS COCO 2017 and PASCAL VOC

The Common Objects in Context (COCO) dataset is a large-scale benchmark developed by Microsoft in collaboration with research institutions. It contains over 330,000 images featuring diverse objects and complex backgrounds and is widely used for computer vision tasks such as object detection, semantic segmentation, and instance-level annotation. Each image is annotated with precise bounding boxes, pixel-level segmentation masks, and corresponding semantic labels. The COCO dataset defines 80 object categories, covering common classes such as humans, animals, and vehicles, while also providing scene-level annotations for background contexts. In the COCO 2017 release, the Train2017 subset (118,287 images) is used for model training, the Val2017 subset (5000 images) for validation, and the Test2017 subset (20,288 images) for performance evaluation and benchmarking.

The PASCAL Visual Object Classes (PASCAL VOC) dataset is a widely used benchmark for object detection, image classification, and semantic segmentation. In this study, the VOC2007 and VOC2012 datasets are integrated to form a combined training set of 21,503 images and a test set of 4952 images, covering a total of 20 object categories.

### 4.2. Experimental Setup

The experimental setup of this study is described as follows. Owing to their larger network architectures and higher parameter counts, YOLOv5X and RT-DETR were applied to complex scenarios in the COCO dataset, whereas YOLOv5L was selected for the PASCAL VOC dataset. Ablation experiments were conducted to assess the performance of RT-DETR on the PASCAL VOC dataset. Training employed stochastic gradient descent (SGD) as the optimizer, with a learning rate of 0.01, momentum of 0.937, and weight decay of 0.0005. Data augmentation techniques included random flipping, rotation, translation, mosaic stitching, and image blending. The label smoothing factor was set to 0.2, and the input image size was fixed at 640 × 640 pixels.

### 4.3. Experimental Analysis

**Figure 5:**
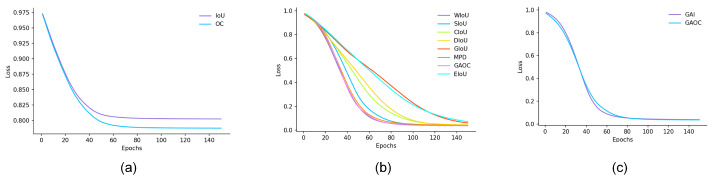
(**a**): IoU and OC loss training for 150 epochs, loss regression. (**b**): Regression curve of the GAOC and other BBR losses. (**c**): Regression curve of the GAOC (OC with GA) and GAI (IoU with GA).

As shown in Figure 5a, under identical initialization conditions, both IoU and OC losses decrease monotonically as training iterations increase, and they reach a plateau after about 50 to 70 epochs, reflecting the stability of the overall optimization process. However, the two curves ultimately converge to loss levels of 0.804 and 0.788, indicating that their localization performance remains limited. By contrast, the OC loss demonstrates a faster descent and a lower convergence value, implying that in cases of minor overlaps or partial scale mismatches, OC provides smoother gradients and thus achieves more effective loss suppression. These results reveal that relying solely on intersection-based metrics (IoU or OC) is insufficient to provide adequate geometric guidance in scenarios with large displacements or significant shape differences.

Based on the results in Figure 5b,c and Table 1, we systematically compared multiple BBR loss functions. As shown in Figure 5b,c, all methods exhibit a stable downward trend in their training curves. However, they differ significantly in convergence speed and final residual values. In terms of curve morphology, WIoU, SIoU, and GAOC decline most rapidly within the 40 to 60 epochs and quickly enter a low loss plateau. GIoU converges the slowest and retains the highest residual error. EIoU descends faster in the mid-to-late training phase but still ends with relatively high residuals. A closer comparison between GAOC (GA + OC) and GAI (GA + IoU) shows that both follow a smooth, S-shaped convergence trajectory. GAOC achieves a lower final value and a more stable plateau. This suggests that it provides effective gradient guidance in both the early geometric alignment and the later fine-tuning stages of localization.

From Table 1, Mean represents the overall average localization quality, whereas Min reflects the lower bound performance on the most challenging samples. Higher values indicate greater robustness. GAOC achieves the best results on both Mean (0.963) and Min (0.956). This demonstrates improvements not only in overall accuracy but also in worst-case long tail performance. GAI (0.959/0.953) yields comparable results. WIoU achieves a higher Min (0.950) than MPDIoU (0.942), indicating stronger robustness. DIoU and CIoU perform at moderate levels. EIoU shows competitive average performance (0.958) but a very low Min (0.51), revealing high vulnerability to long tail cases. GIoU performs the weakest across both metrics (0.872/0.571).

Mechanistically, GAOC and GAI integrate overlap measures with Gaussian corner-based geometric constraints. These constraints yield nonvanishing, directionally informative gradients even under zero overlap and weak overlap conditions. This design enables fast convergence, low residual loss, and strong long tail robustness. WIoU mitigates noisy gradient effects through hard sample adaptive weighting, thereby stabilizing its tail performance. In contrast, EIoU suffers from gradient imbalance when dealing with extreme shapes and large displacements. This results in degraded tail performance. In summary, GAOC achieves the best overall performance in this simulation study, excelling in convergence speed, final accuracy, and long tail robustness.

Table 2 and Table 3 present the experimental results on the COCO 2017 and PASCAL VOC datasets, respectively. A comparison of different BBR losses (including CIoU, DIoU, EIoU, GIoU, SIoU, WIoU, and MPDIoU) against GAOC yields the following conclusions. On the COCO 2017 dataset, GAOC outperformed all competing methods in mAP, mAP75, and mAP50. Specifically, GAOC achieved an mAP of 46.2%, representing a 1.6% improvement over the best-performing competitor, WIoU (44.6%), thereby demonstrating a clear advantage in detection accuracy. GAOC also achieved an mAP50 of 64.5%, corresponding to a 3.2% increase over WIoU and substantial improvements compared to other methods.

**Figure 6:**
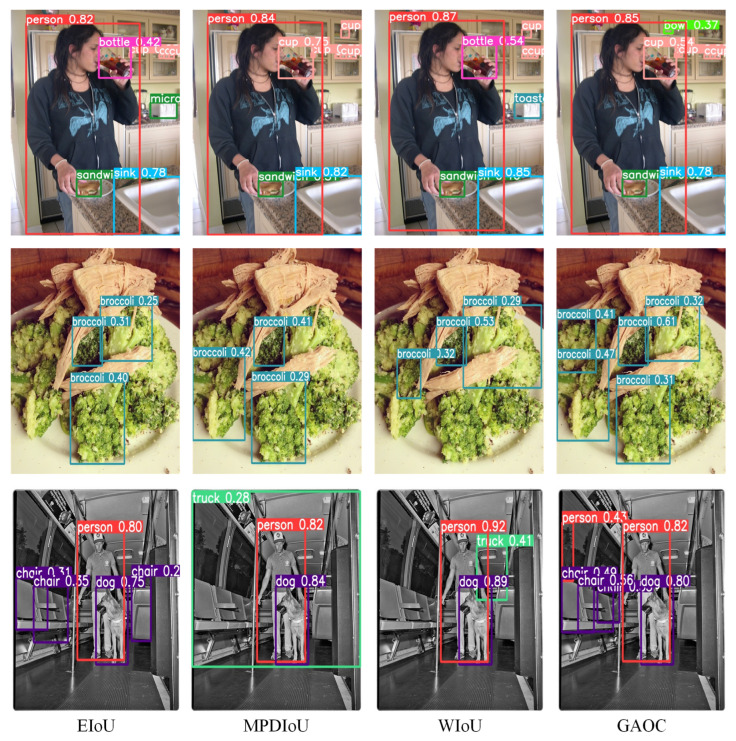
Inference performance comparison of various BBR losses on COCO.

**Figure 7:**
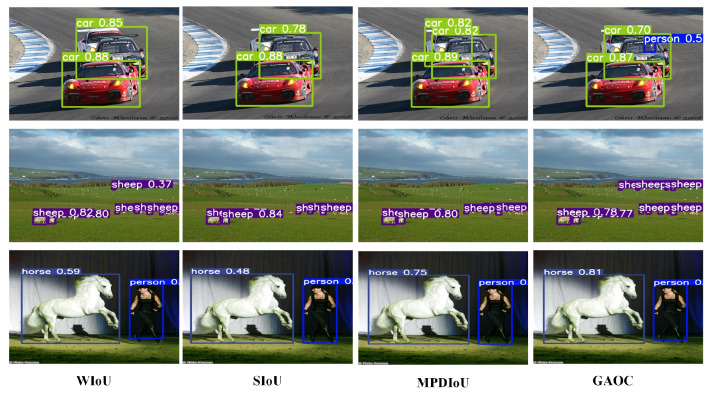
Inference performance comparison of various BBR losses on PASCAL VOC.

On the PASCAL VOC dataset, GAOC achieved an mAP50 of 79.0%, further confirming its superior performance. The consistent improvements across both datasets highlight GAOC’s robust generalization capability: it sustains high detection accuracy in the complex, real-world scenes of COCO 2017 as well as in the standardized image settings of PASCAL VOC. Figure 6 and Figure 7 illustrate qualitative detection results, further supporting the superior performance of GAOC compared with alternative loss functions.

Table 4 presents the performance of various BBR losses functions on the MS COCO dataset using RT-DETR as the baseline, with GAOC demonstrating clear advantages across key evaluation metrics. For the mAP50 metric, GAOC achieved the highest score of 65.3%, surpassing the second-ranked CIoU (64.9%) by 0.4%. This result indicates that GAOC delivers superior object detection accuracy under relaxed matching criteria. GAOC also maintained the leading position in mAP, achieving a score of 47.8%. Overall, in experiments on the MS COCO dataset with RT-DETR as the benchmark, GAOC consistently outperformed other mainstream BBR losses functions (including CIoU, DIoU, and EIoU) across both mAP50 and mAP metrics. These findings confirm that GAOC more effectively optimizes BBR in object detection, improves detection accuracy, and provides superior overall performance.

The design philosophy of GAOC is grounded in a deep understanding of the intrinsic characteristics of the BBR problem. By introducing a falloff coefficient, GAOC assigns greater weight to the intersection between predicted and ground truth boxes in the loss function, thereby improving detection performance for multi-scale objects. In addition, the incorporation of a Gaussian adaptive mechanism increases the algorithm’s robustness to variations in target position by modeling the TL/BR coordinates of bounding boxes as a two-dimensional Gaussian distribution.

Synthesizing the experimental results with the methodological analysis, we conclude that the GAOC loss function demonstrates outstanding performance in object detection tasks. The combination of its innovative design and consistent empirical outcomes validates GAOC as a novel BBR loss function with significant potential to advance the field of object detection. Future research could explore applying GAOC to diverse scenarios and tasks, as well as integrating it with other state-of-the-art (SOTA) algorithms.

### 4.4. Ablation Study

When comparing the OC and IoU loss functions, as shown in Table 5, OC consistently outperforms IoU across both mAP50 and mAP. By normalizing the square root of bounding box dimensions’ product, OC achieves scale invariance and enables more accurate similarity measurement between predicted and ground truth boxes, thereby delivering superior performance in object detection tasks.

The comparison between GAI and GAOC shows that GAOC surpasses GAI by 1.2% and 0.5% in mAP50 and mAP, respectively. This result validates the rationale for integrating GA with OC. The GA mechanism reduces sensitivity to positional deviations by modeling the TL/BR coordinates of bounding boxes as a two-dimensional Gaussian distribution. The scale invariance of OC further complements this approach. In contrast, GAI’s reliance on IoU limits its optimization capacity, and even when combined with GA, it fails to match the comprehensive performance of GAOC. These findings suggest that GAOC adopts a more effective design strategy for BBR optimization, better addressing the challenges of complex scenes and multi-scale object detection.

## 5. Conclusions

This study proposed the GAOC loss function. Experimental results on the COCO 2017 and PASCAL VOC benchmark datasets demonstrated that GAOC delivers significant performance improvements in object detection tasks. GAOC enhances the detector’s capability for multi-scale object detection and reduces sensitivity to positional bias. It achieves this by assigning greater weight to the intersection of predicted and ground truth boxes and implementing point-to-point coordinate alignment. These experiments not only validate the effectiveness of GAOC but also highlight its strong potential for practical applications. Future work may extend GAOC to related domains, such as instance segmentation.

## Figures and Tables

**Table 1:** Performance of each bounding box loss.

	Mean	Min
WIoU	0.958	0.950
SIoU	0.956	0.944
CIoU	0.946	0.848
DIoU	0.948	0.869
GIoU	0.872	0.571
MPDIoU	0.958	0.942
EIoU	0.958	0.51
GAI	0.959	0.953
GAOC	0.963	0.956

**Table 2:** Performance of each bounding box loss on the COCO with YOLOv5X as the baseline.

	mAP	mAP75	mAP50	mAP_*s*_	mAP_*m*_	mAP_*l*_
CIoU	43.1	46.6	60.8	26.5	47.9	56.0
DIoU	42.6	46.1	60.0	25.8	47.3	56.1
EIoU	44.1	48.1	60.7	27.4	50.2	57.2
GIoU	42.4	45.9	59.7	26.2	46.9	55.7
SIoU	42.5	46.6	60.0	26.1	47.1	55.5
WIoU	44.6	48.5	61.4	28.1	50.0	58.3
MPDIoU	44.5	48.5	61.2	27.8	50.4	57.4
GAOC	46.2	50.0	64.5	28.5	40.8	60.6

**Table 3:** Performance of each bounding box loss on the PASCAL VOC with YOLOv5L as the baseline.

	mAP	mAP75	mAP50
CIoU	59.4	65.4	78.3
DIoU	59.5	65.6	78.1
EIoU	59.5	65.4	78.6
GIoU	59.2	64.9	78.1
SIoU	59.7	65.7	78.4
WIoU	59.6	65.3	78.4
MPDIoU	59.5	65.2	78.5
GAOC	60.2	66.2	79.3

**Table 4:** Performance of each bounding box loss on the MS COCO with RT-DETR as the baseline.

	mAP50	mAP
CIoU	64.9	47.4
DIoU	64.3	47.0
EIoU	61.1	44.3
GIoU	64.7	47.3
SIoU	64.1	46.7
WIoU	63.6	46.4
MPDIoU	63.8	45.9
GAOC	65.3	47.8

**Table 5:** The ablation experiments were conducted on the PASCAL VOC dataset using RT-DETR as the baseline model to evaluate the contribution of each loss component.

	mAP50	mAP
IoU	70.9	52.2
OC	71.7	52.4
GAI	72.6	53.1
GAOC	73.8	53.6

## Data Availability

All datasets used in this study are publicly available and ethically compliant. There are no competing interests associated with the data. The code used and analyzed during the current study is available from the corresponding or first author upon reasonable request.
